# Transition of oral microbiome profile in HIV-infected Indonesian patients: the role of antiretroviral therapy

**DOI:** 10.1080/20002297.2025.2609445

**Published:** 2026-01-02

**Authors:** Muhammad Anshory, Nikolaos Strepis, John P. Hays, Milanitalia Gadys Rosandy, Aulia Rahmi Prawesti, Agustin Iskandar, Indah Adhita Wulanda, Lita Setyowatie, Nathanael Ibot David, Natalia Rasta Malem, Handono Kalim, Tamar E. Nijsten, Jan L. Nouwen, Hok Bing Thio

**Affiliations:** a Department of Dermatology, Erasmus University Medical Centre (Erasmus MC), Rotterdam, The Netherlands; b Department of Internal Medicine, Faculty of Medicine, Universitas Brawijaya, Malang, Indonesia; c Department of Pathology, Erasmus University Medical Centre (Erasmus MC), Rotterdam, The Netherlands; d Department of Medical Microbiology and Infectious Diseases, Erasmus University Medical Centre (Erasmus MC), Rotterdam, The Netherlands; e Department of Parasitology, Faculty of Medicine, Universitas Brawijaya, Malang, Indonesia; f Department of Clinical Pathology, Faculty of Medicine, Universitas Brawijaya, Malang, Indonesia; g Department of Dermatology and Venereology, Faculty of Medicine, Universitas Brawijaya, Malang, Indonesia

**Keywords:** Oral microbiome, HIV, antiretroviral therapy (ART), dysbiosis, 16S rRNA sequencing

## Abstract

**Background:**

HIV infection alters host immunity, including the oral environment, leading to microbial imbalance and increased risk of opportunistic infections. Although antiretroviral therapy (ART) improves immune function, its effect on the oral microbiome remains unclear, particularly in Indonesia. This study investigated oral microbiome composition in people living with HIV and its associations with ART status, age, and sex.

**Methods:**

In this cross-sectional study, oral rinse samples from 245 adults (115 HIV-on-ART, 15 HIV-ART-naïve, and 115 HIV-negative controls) were analysed using 16S rRNA gene sequencing. Alpha and beta diversity metrics, differential abundance (ANCOM-BC2), and multivariable associations (PERMANOVA) were assessed.

**Results:**

The oral microbiome differed significantly between HIV-positive groups and controls (PERMANOVA *p* = 0.001, R² = 1.8%). HIV-ART-naïve individuals exhibited the highest alpha diversity and enrichment of pro-inflammatory genera, including *Fusobacterium*, *Alloprevotella,* and *Staphylococcus*. ART-treated individuals displayed a partial shift toward the control profile but retained persistent depletion of bacteria such as *Filifactor* and *(Eubacterium) saphenum*. Multivariate analysis identified HIV status, age, and sex as independent contributors to microbial variation.

**Conclusion:**

HIV infection is associated with a distinct oral dysbiosis characterised by an increase in opportunistic pathogens and reduction in commensal bacteria. HIV-on-ART individuals show a transitional shift towards the HIV-negative oral microbiome profiles. Our findings suggest that biological and/or demographic factors coupled to oral microbiome profiles may facilitate targeted interventions in the personalised management of oral health for individuals living with HIV.

## Introduction

The human oral cavity hosts a rich and intricate community of microorganisms, collectively known as the oral microbiome, which includes bacteria, archaea, fungi, and viruses that are frequently incorporated within biofilms [1]. By maintaining a balanced ecosystem, this microbiome plays a crucial role in supporting oral homeostasis and is increasingly recognised as having an impact on overall systemic health [2]. In fact, growing attention is being given to how the composition of the oral microbiome can contribute to diseases such as chronic inflammation in the oral cavity (e.g. periodontitis), and how these diseases may influence distant body sites, potentially contributing to systemic diseases [3].

A prime example of this systemic interplay is observed in individuals with Human Immunodeficiency Virus (HIV). HIV infection is known to create persistent viral reservoirs in various tissues and to significantly modulate the composition of host microbial communities [4,5]. Specifically, HIV often compromises local immune function, disrupting the delicate balance that helps maintain an individual’s commensal oral microbiome [6]. This weakened immunity (characterised by decreased salivary enzymes, protein deficiencies, and the depletion of immune cells) can lead to the development of HIV-related conditions and other non-AIDS comorbidities [7].

The rationale for understanding these oral health complications is underscored by the ongoing global HIV epidemic. Although infection rates are declining worldwide, HIV remains a significant challenge in countries like Indonesia, where new infections continue to rise, where an estimated 570,000 people are living with the virus, mainly in the regions of Tanah Papua and East Java. Of pressing public health concern is the HIV-treatment gap, as only 31% of infected individuals receive antiretroviral therapy (ART) and a mere 14% achieve viral suppression [8]. ART suppresses viral replication, lowers plasma HIV RNA levels, and promotes immune recovery through the restoration of CD4+ T cells [9]. However, residual immune dysfunction and chronic inflammation may persist despite effective therapy, potentially affecting host–microbiome balance [4]. Therefore, the HIV epidemic is a major burden on healthcare systems in Indonesia, making the investigation of factors that influence overall health, such as the state of the oral microbiome, a high priority [8,10].

Therefore, the aim of this study was to characterise differences in the composition of the oral microbiome between HIV-ART-Naïve, HIV-on-ART, and HIV-negative individuals living in Indonesia, and to correlate these findings to demographic data obtained from these three groups.

## Methodology

### Study design and participants

This was a prospective, observational study, designed to conduct a comparison of clinical characteristics and the composition of the oral microbiome in three distinct participant groups. The research was conducted at the outpatient and inpatient settings of the Dr. Saiful Anwar Regional General Hospital in Malang, Indonesia. Participants aged over 17 years were recruited using a consecutive random sampling method and were divided into three groups:

1. HIV-ART-naïve group: This group included newly diagnosed HIV-positive patients (WHO stages 1-4), who had not yet initiated antiretroviral therapy (ART) or had received therapy for less than two weeks. Patients who had previously received ART but had discontinued the therapy for more than six months, were also eligible for inclusion in this group.
2. HIV-on-ART group: This group consisted of HIV-positive patients who had been consistently receiving ART for a minimum of one year.
3. HIV-Negative Control group: This group consisted of control individuals recruited from general population, without HIV infection at the time of enrolment, serving as a baseline for comparison.

### Study period and ethical considerations

This research was conducted in accordance with ethical standards. The study protocol first received full approval from the institutional ethics committee at Dr. Saiful Anwar General Hospital, Malang (reference number 400/135/K.3/102.7/2023, 5 June 2023). Participant recruitment and data collection took place from October 2023 through June 2024. Each participant's involvement began only after they received a thorough explanation of the research and voluntarily gave their written informed consent. Strict data privacy was ensured via a secure, access-restricted, electronic system for all questionnaire data. With respect to international collaboration, the shipment of all biological specimens to Erasmus MC was formally authorised through a Material Transfer Agreement (MTA), which was reviewed and overseen by the Indonesian Ministry of Health's Health Development Policy Agency (BKPK Kemenkes RI) reference number FK.06.01/H/5614/2024.

### Data and sample collection procedures

All collection procedures were standardised and performed by trained research personnel to ensure consistency and data integrity.

#### Clinical and demographic data collection

A detailed medical history (anamnesis) and demographic information were collected from each participant using a structured, validated electronic questionnaire. These demographic and socioeconomic variables were collected to comprehensively characterise the study population and were also evaluated as potential determinants of oral microbiome variation in univariate and multivariate PERMANOVA analyses. The collected variables included:

- General Parameters: Age, sex, occupation, marital status, education, income, date of HIV diagnosis, and putative mode of HIV transmission. Data on oral hygiene practices and any presenting complaints were also recorded. Oral health hygiene behaviour was assessed using a structured, previously validated, oral health habit questionnaire designed to capture each participant’s routine oral care practices [11]. The questionnaire included items on frequency of toothbrushing per day, nighttime toothbrushing per week, mouthwash use, flossing, and dental scaling frequency. Responses were combined to generate a ‘Total Oral Health Habit Score,’ reflecting overall hygiene consistency (see Supplementary Data 2 for the complete scoring system).
- Clinical Examination: A comprehensive physical and oral examination was performed by specialist physicians (dermatologists) from the department of dermatology and venereology. All cutaneous and oral lesions were diagnosed and documented, specifying their type and anatomical location. As the assessment was designed to focus on dermatologic and mucosal findings rather than dental pathology, detailed dental parameters (for example, plaque status, caries, and periodontal measurements) were not included. The primary diagnosis, any comorbidities, and the level of care (outpatient vs. inpatient) were recorded.
- Treatment History: A complete record of all current and past pharmacotherapies was documented, including treatments for HIV and other comorbidities.

#### Blood sample collection and laboratory analysis

A venous blood sample was drawn from each participant by a certified phlebotomist. The samples were subsequently transported to the hospital's central laboratory for CD4+ T-cell count analysis.

#### Oral microbiome sample collection

The collection of the oral microbiome was performed through an oral rinse, using a protocol adapted from a previous study [12]. Each participant was instructed to vigorously swish and rinse for a full 60 seconds with 15 mL of sterile Phosphate-Buffered Saline (PBS) before collecting the entire expectorant into a sterile 50 mL conical tube. To maintain sample integrity, the collected rinse was immediately placed on ice for prompt processing in the laboratory. There, the sample was subjected to centrifugation at 2,000 x g for 10 minutes, a step designed to concentrate the microbial cells into a dense pellet. Following this, the supernatant was carefully decanted, leaving the cell pellet undisturbed in a small amount of residual liquid. This concentrated pellet was then thoroughly resuspended in 1 mL of COPAN eNAT® (Copan Italia s.p.a., Italy) medium and stored at −20 °C pending DNA extraction.

## Microbiome processing and analysis

### DNA extraction

DNA extraction from oral samples was performed at the Laboratory of Parasitology, Faculty of Medicine, Brawijaya University, Malang. The QIAamp DNA Microbiome Kit (QIAGEN, Netherlands) was used to preferentially isolate microbial DNA while minimising host DNA contamination. The procedure involved initial sample preparation with benzonase to degrade host DNA, followed by mechanical lysis using bead beating to disrupt microbial cell walls. Subsequent steps included protein digestion with proteinase K, DNA binding to a silica spin column, washing with AW1 and AW2 buffers to remove inhibitors, and final elution of purified DNA in 50 μL of buffer AUE. Extracted DNA was stored at −20 °C.

### Sample shipment and sequencing

All extracted DNA samples were shipped to the Erasmus University Medical Centre (Erasmus MC) in Rotterdam, the Netherlands, for sequencing and bioinformatics analyses. Samples were transported via a professional international courier in a cryobox equipped with a freezer and a temperature logger to ensure a stable −20 °C was maintained throughout transit.

After receipt, the Department of Clinical Genetics (Genomics Core Facility) of Erasmus MC performed Next-Generation Sequencing (NGS) using the 16S rRNA V3-V4 gene region following these 4 steps:

1. 16S rRNA Gene Amplification: The hypervariable V3-V4 region of the 16S rRNA gene was amplified from the extracted DNA using universal primers in a Polymerase Chain Reaction (PCR).
2. Library Preparation: Molecular barcodes were added to the amplicons of each sample to allow for multiplexing (pooling and sequencing multiple samples simultaneously).
3. DNA Cleanup: The amplified DNA library was purified using magnetic beads to remove primers, dNTPs, and non-target DNA fragments.
4. Sequencing: The prepared library was sequenced using an Illumina NextSeq2000 high-throughput sequencing platform (Illumina, San Diego, CA, U.S.A.) to generate millions of short DNA reads.

### Statistical analysis

Raw sequencing reads were initially processed at Erasmus MC using a DADA2 bioinformatics pipeline for quality control, read filtering, and taxonomic classification. The database used for taxonomic assignment was the SILVA 16S database release 138.1 [13]. The resulting data, containing amplicon sequence variant (ASV) counts and classifications, were imported into R (v. 4.5.0) and merged with sample metadata containing demographic and clinical variables.

Group comparisons for clinical and demographic variables were performed using the χ2 (Chi-square) test for categorical data. For continuous variables, we selected either the independent t-test/ANOVA or the Mann-Whitney U test following an assessment of data normality. To analyse the microbial community composition, counts were normalised to relative abundance. We then assessed beta diversity (between-sample dissimilarity) using the Bray-Curtis metric and visualised the community composition using Principal Coordinates Analysis (PCoA). The statistical significance of differences in the overall microbial composition between groups was tested using a Permutational Multivariate Analysis of Variance (PERMANOVA) with 999 permutations.

A rigorous decontamination and filtering workflow was first implemented to ensure data integrity. Contaminant ASVs were identified and removed using the decontam R package [14], which flags taxa disproportionately found in negative controls or correlated with low DNA concentrations. Subsequently, a blacklist of contaminant genera was used to filter out any unwanted microbial sequences from the dataset [15]. The dataset was further curated by removing rare taxa (present in under 10% of samples), ambiguous or unclassified taxonomic assignments, and any samples with zero remaining reads. This multi-stage process yielded a high-quality dataset focused on biologically relevant microbial signals.

To pinpoint any specific taxa-driving community differences, we performed a differential abundance analysis with ANCOM-BC2, which identifies microbes with altered abundances while accounting for the compositional nature of the data and providing bias-corrected statistical inference. Key findings were visualised with waterfall plots and heatmaps of log-fold change (LFC) abundances. Finally, to disentangle the complex relationships between host factors and the microbiome, we implemented a PERMANOVA model to simultaneously assess the influence of demographic and clinical parameters. The final multivariable model included age, sex, BMI, CD4+ T-cell count, HIV or ART group, and the Total Oral Health Habit score. Socioeconomic variables (marital status, education, income) were excluded from the final model because they lacked direct biological justification and did not materially influence model performance. The entire analytical workflow was executed using R (v. 4.5.0) utilising packages such as phyloseq, vegan, ANCOMBC, and ggplot2.

## Results

### Baseline characteristics

Oral microbiome samples were collected from 245 participants comprising 130 HIV-positive participants, where 115 were receiving ART (‘HIV-on-ART’ group) and 15 not receiving ART (‘HIV-ART-naïve’ group). The remaining 115 were non-HIV infected healthy controls (‘Control’ group). The average ages were 42 years for the HIV-on-ART group, 32 years for the HIV-ART-naïve group and 40 years for the controls. There was a lower proportion of women in both HIV groups, with 46.7% in the HIV-ART-naïve group and 41.7% in the HIV-on-ART group, compared to 53.9% women in the control group, though these differences were not statistically significant (*p* = 0.181). Complete baseline characteristics are provided in Table 1.

**Table 1.** Baseline characteristics of study subjects.

	HIV-ART-naïve	HIV-on-ART	Control	
n	15	115	115	*p*
Age; mean (SD)	31.80 (7.79)	42.31 (10.50)	39.98 (11.19)	0.001
Sex = Women; *n* (%)	7 (46.7)	48 (41.7)	62 (53.9)	0.181
Marriage status; *n* (%)				0.014
Single	6 (40.0)	31 (27.0)	24 (20.9)	
Married	9 (60.0)	61 (53.0)	83 (72.2)	
Divorcee	0 (0.0)	12 (10.4)	3 (2.6)	
Widowed	0 (0.0)	11 (9.6)	5 (4.3)	
Education; *n* (%)				<0.001
Elementary School	4 (26.7)	25 (21.7)	7 (6.1)	
Junior High School	1 (6.7)	12 (10.4)	12 (10.4)	
Senior High School	7 (46.7)	51 (44.3)	90 (78.3)	
Bachelor's or equivalent	3 (20.0)	25 (21.7)	6 (5.2)	
Postgraduate	0 (0.0)	2 (1.7)	0 (0.0)	
Income; in Indonesian Rupiah (IDR); *n* (%)				0.034
<IDR 500.000	5 (33.3)	13 (11.3)	7 (6.1)	
IDR 500.000−2.500.000	4 (26.7)	63 (54.8)	71 (61.7)	
IDR 2.500.000−5.000.000	5 (33.3)	31 (27.0)	34 (29.6)	
IDR 5.000.000−10.000.000	1 (6.7)	6 (5.2)	3 (2.6)	
>IDR 10.000.000	0 (0.0)	2 (1.7)	0 (0.0)	
Body Mass Index; kg/m^2^; mean (SD)	23.16 (3.40)	22.07 (4.33)	24.26 (4.17)	0.001
Haemoglobin level; gr%; mean (SD)	11.99 (2.23)	13.43 (2.03)	14.20 (1.88)	<0.001
CD4+ T-cell;/μL; mean (SD)	266.91 (118.95)	548.46 (270.98)	904.03 (288.21)	<0.001
Total Oral Health Habit score; mean (SD)	2.87 (1.41)	3.57 (2.09)	3.47 (1.88)	0.420
HIV disease duration; mean (SD)	2.33 (3.15)	9.10 (4.52)	**–**	<0.001
Antiretroviral therapy (ART) duration; mean (SD)	1.00 (0.00)	8.78 (4.51)	**–**	0.004

### Microbial community structure

#### Alpha diversity analysis

**Figure 1.**
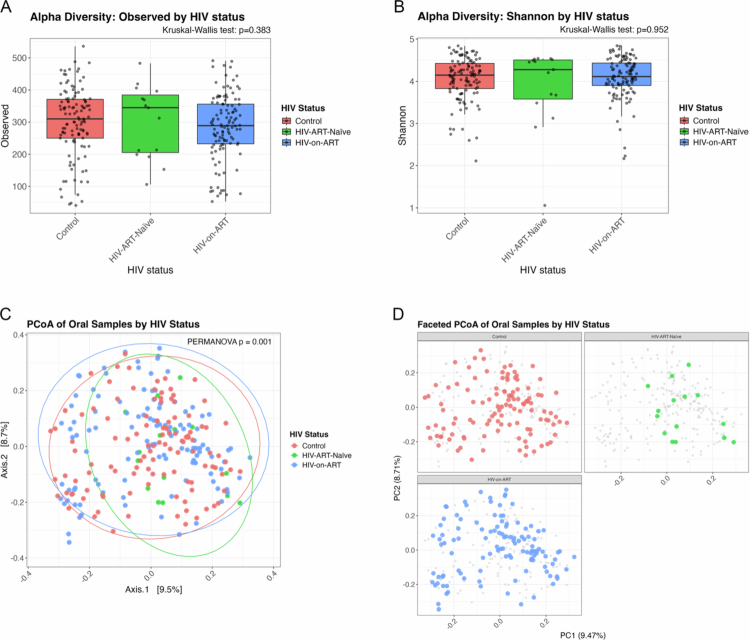
Microbial diversity and community structure across study groups. (A) Alpha diversity measures by Observed species (richness) and (B) Shannon index (richness and evenness) across healthy controls (orange), HIV-ART-naïve (green), and HIV-on-ART (blue) groups. Boxplots display the median, interquartile range (IQR; 25th–75th percentile), and whiskers extending to 1.5 × IQR, with individual points representing each sample. No significant differences were detected by Kruskal–Wallis test (Observed: *p* = 0.383; Shannon: *p* = 0.952). (C) Principal Coordinates Analysis (PCoA) of Bray-Curtis distances (beta diversity) showing overall oral microbiome community composition by group. Each point represents an individual sample, with ellipses indicating 95% confidence intervals. PERMANOVA demonstrated significant differences among groups (*p* = 0.001). (D) Faceted PCoA plots illustrating group-specific clustering of oral microbiome. Healthy control (orange), HIV-ART-naïve (green), and HIV-on-ART (blue).

Alpha diversity indices (Observed species and Shannon) were compared across the three groups to evaluate microbial richness and evenness within each sample (Figure 1A,B). The Observed Species index (number of species) revealed a higher median richness in the HIV-ART-naïve group, compared to HIV-on-ART and controls, although this difference was not statistically significant (Kruskal–Wallis *p* = 0.383) (Figure 1A). The Shannon index, which reflects both richness and evenness, showed a similar trend, with HIV-ART-naïve individuals tending to have higher diversity, though again without statistical significance (Kruskal–Wallis *p* = 0.952) (Figure 1B). Therefore, we did not find evidence that the alpha diversity of the oral microbial is significantly altered by HIV status or antiretroviral therapy.

#### Beta diversity analysis

To explore differences in overall microbial community composition, Principal Coordinate Analysis (PCoA) based on Bray-Curtis dissimilarity was performed. PERMANOVA confirmed that overall microbial composition has a statistically detectable difference among the three groups (*p* = 0.001), although the effect size was very small (R^2^ = 0.018). Consistent with the substantial overlap on the PCoA plot, these results show that while a group-level difference is statistically detectable, it accounts for very little community variation, and the groups remain highly similar.

In the combined PCoA plot, the HIV-ART-naïve group formed a more constrained cluster, suggesting a relatively homogeneous microbial composition and unique microbial composition. In contrast, samples from the HIV-on-ART and control groups displayed broader and partially overlapping distributions, indicating greater inter-individual variation and community similarity between these two groups (Figure 1C). Faceted PCoA plots further emphasised this conclusion. The HIV-ART-naïve, HIV-on-ART and control groups showed dispersed and overlapping clustering (Figure 1D). Overall, these findings confirm considerable similarity in community structure across groups despite statistically detectable differences.

#### Differential abundance analysis

**Figure 2.**
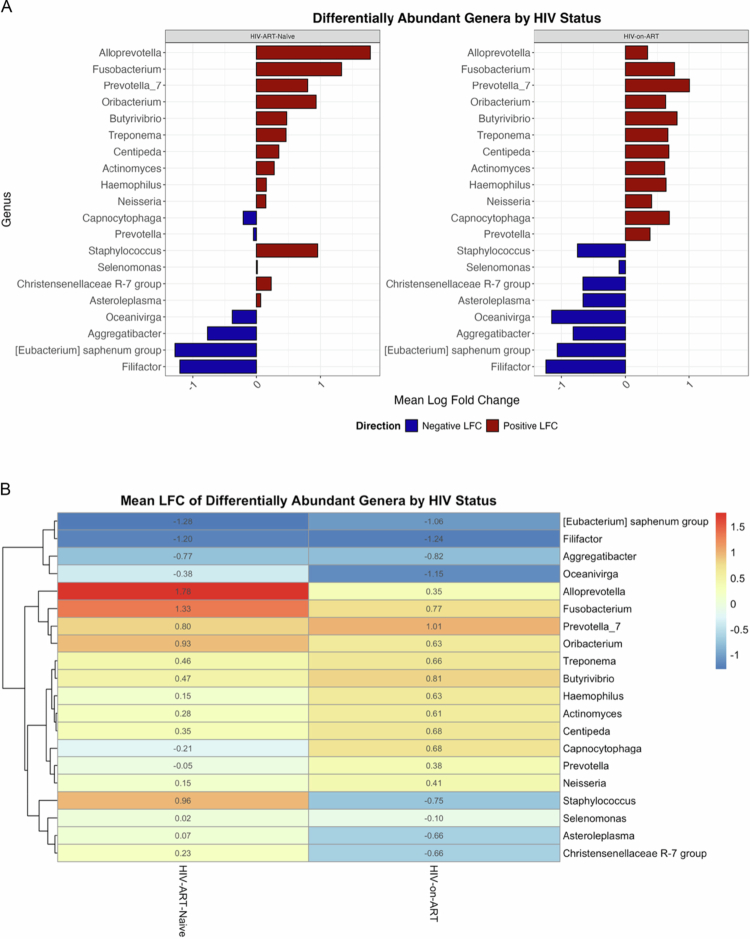
Differentially abundant oral bacterial genera in HIV groups compared to healthy controls. (A) Waterfall plot showing Log Fold Change (LFC) for 19 genera with significant differences in abundance in the HIV-ART-naïve (left) and HIV-on-ART (right) groups relative to healthy controls (ANCOM-BC, q < 0.05). (B) Heatmap displaying the mean LFC for the same genera in HIV-ART-naïve and HIV-on-ART groups. Colour scale indicates enrichment (red) or depletion (blue) relative to controls.

Using the ANCOM-BC method, we conducted a genus-level differential abundance analysis to evaluate the impact of HIV infection and ART on the oral microbiome. A total of 20 genera were found to be significantly altered (*p* < 0.05) in at least one of the comparison groups (Figure 2A, B). In HIV-ART-naïve individuals compared to healthy controls, thirteen genera were significantly enriched, most notably *Alloprevotella* (LFC = +1.78), *Fusobacterium* (LFC = +1.33), and *Staphylococcus* (LFC = +0.96). Other enriched genera included *Treponema, Centipeda, Oribacterium*, and *Prevotella_7*. Conversely, seven genera were significantly depleted in HIV-ART-naïve individuals relative to healthy controls. The most strongly depleted taxa were *(Eubacterium) saphenum* group (LFC = –1.28), *Filifactor* (LFC = –1.20), and *Aggregatibacter* (LFC = –0.77).

In the HIV-on-ART group (Figure 2A, B), genera such as *Fusobacterium* (LFC = +0.77), *Alloprevotella* (LFC = +0.35), and *Prevotella_7* (LFC = +1.01) were significantly enriched compared to controls. In contrast, other taxa were found to be depleted. *Staphylococcus*, for example, was significantly depleted in the HIV-on-ART group (LFC = –0.75) as were *Christensenellaceae R-7* group (LFC = +0.23 in HIV-ART-naïve vs. –0.66 in HIV-on-ART) and *Asteroleplasma* (LFC = + 0.07 in HIV-ART-naïve vs. –0.66 in HIV-on-ART).

Nevertheless, the depletion of several health-associated genera persisted despite ART. *Filifactor* (LFC = –1.24), *(Eubacterium) saphenum* group (LFC = –1.06), and *Aggregatibacter* (LFC = –0.82) remained significantly underrepresented in the HIV-on-ART group compared to healthy controls.

### Identifying key drivers of oral microbiome variation

A multivariate PERMANOVA was conducted to explore determinants of oral microbiome composition (Table 2). After adjusting for age, sex, BMI, CD4+ T-cell count, HIV or ART status, and oral health behaviour, HIV status remained a significant predictor of community composition (R² = 0.013, *p* = 0.003). Age (*p* = 0.001) and sex (*p* = 0.002) also emerged as significant contributors, although with small effect sizes, whereas BMI, CD4+ T-cell count, and the Total Oral Health Habit score were not significantly associated with overall community variation.

**Table 2.** PERMANOVA analysis.

Variables	Univariate			Multivariate model (*n* = 227)		
	R^2^	F	*p*	R^2^	F	*p*
Age	0.013	3.316	0.001	0.011	2.549	0.001
Sex	0.010	2.376	0.002	0.009	2.067	0.002
Marriage	0.012	0.997	0.442			
Education	0.021	1.271	0.023			
Income	0.017	1.007	0.421			
Body Mass Index	0.005	1.240	0.131	0.004	0.943	0.532
CD4+ T-cell count	0.010	2.273	0.001	0.005	1.071	0.299
Total Oral Health Habit score	0.004	0.998	0.444	0.003	0.768	0.859
HIV Status	0.0185	2.274	0.001	0.013	1.526	0.003

In univariate PERMANOVA, CD4+ T-cell count showed a small but statistically significant association with beta diversity (R² = 0.010, *p* = 0.001); however, this effect disappeared in the multivariable model, suggesting that CD4-related variation is largely explained by other covariates. To further clarify this relationship, we conducted a separate analysis restricted to HIV-positive participants (Supplementary Data 3). Within this subgroup, age was the only variable that remained significantly associated with microbial composition in the multivariable model (R² = 0.018, *p* = 0.001), while CD4+ T-cell count again showed no independent effect.

### Sex- and age-associated microbial signatures

Differential abundance analysis using ANCOM-BC revealed distinct sex- and age-specific patterns in oral bacterial composition (Supplementary Figure 1 A). These models used the combined three-level group variable (‘Control’, ‘HIV-ART-naïve’, ‘HIV-on-ART’) as the main formula and applied Holm’s correction at the ASV level, with significant ASVs aggregated to genus level for visualisation. In the overall cohort (i.e. all three groups combined), men exhibited significantly higher relative abundances of *Lactobacillus, Weissella, (Eubacterium) brachy* group*, Megasphaera,* and *Stomatobacterium*, with LFC ranging from approximately +0.75 to +1.0. Additional taxa such as *Phocaeicola, Streptobacillus, and (Eubacterium) saphenum* group were also more abundant in men. In contrast, women were enriched in *Moraxella, Haemophilus, Fusobacterium, Filifactor,* and *Anaeroglobus,* with *Moraxella* showing an LFC exceeding +1.5 and the others ranging between +0.75 and +1.0. Several additional genera, including *Actinobacillus, Rothia, Porphyromonas, Bergeyella, Prevotella, Alloprevotella, (Eubacterium) nodatum group,* and *Neisseria,* were also notably enriched in women, though these genera were less abundant overall.

Within the HIV-on-ART subgroup (Supplementary Figure 1B), sex-associated microbial compositions differed from those observed in the overall cohort. Men exhibited higher levels of *Weissella, Eikenella, Lactobacillus, Haemophilus,* and *Olsenella,* whereas women were enriched in *Fusobacterium, (Eubacterium) nodatum group, Actinobacillus, Centipeda,* and *Treponema.*

Age was also associated with significant directional shifts in specific genera (Supplementary Figure 1 C). In the full cohort, older age correlated with increased abundance of *Filifactor* (+0.08 LFC/year), *Rothia* (+0.07), *Limosilactobacillus* (+0.05), and *Actinomyces* (+0.05), while genera such as *Haemophilus* (–0.06 LFC/year), *Alloprevotella* (–0.05), *Porphyromonas* (–0.05), and *Prevotella_7* (–0.02) decreased significantly with age. Within the HIV-on-ART subgroup, *Limosilactobacillus, Rothia,* and *Lactobacillus* exhibited more pronounced age-related increases (up to +0.1 LFC/year), while *Alloprevotella* exhibited a marked decline, exceeding −0.15 LFC per year (Supplementary Figure 1D).

A parallel sub-analysis was also attempted in the HIV-ART-naïve group, although an insufficient sample size meant that the ANCOM-BC model failed to converge. This resulted in non-estimable (NA) outputs, precluding meaningful interpretation of sex- or age-related microbial differences in this subgroup.

## Discussion

In this study, HIV infection was associated with distinct shifts in the oral microbiome, characterised by enrichment of opportunistic, pro-inflammatory associated pathogens and the reduced abundance of bacterial taxa considered to be commensal or beneficial [16]. This finding is in line with current research, which suggests that HIV-associated oral dysbiosis is characterised by a replacement of key bacterial taxa, rather than a collapse in overall community complexity, leading to an altered yet equally diverse ecosystem [7].

Among individuals receiving ART, oral microbiome profiles tended to move closer to those of HIV-negative controls, although several HIV-negative-associated genera remained depleted, and the overall community composition remained distinct. Beta diversity calculations provided a community-level view of this dysbiosis, with Principal Coordinate Analysis (PCoA) showing a significant, but small, difference in the oral microbiome composition between the HIV-ART-naïve group and the control group. Interestingly, these shifts in community composition were not accompanied by changes in alpha diversity, as we observed no significant differences in species richness (Observed Species) or overall diversity (Shannon index) among the three groups. This finding supports the idea that HIV-associated dysbiosis involves compositional replacement rather than loss of overall diversity, a finding that aligns with prior reports indicating that ART does not markedly reduce overall diversity, but rather promotes a shift in microbial composition toward an HIV-negative profile [17,18]. This shift may be a consequence of improved immune status and a partial reduction in chronic inflammation, rather than a direct effect of ART on the microbiome *per se* [17].

### Immune status and community-level microbiome variation

In multivariable PERMANOVA analyses, CD4+ T-cell count was not independently associated with oral microbiome composition after adjustment for age, sex, HIV or ART status, and oral health behaviour, despite showing a small effect in univariate models. This suggests that CD4-related variation largely reflects broader host and disease characteristics rather than a direct influence of current peripheral immune status. Consistent with this interpretation, analyses restricted to HIV-positive participants identified age as the only independent determinant of community composition, with no residual effect of CD4+ T-cell count. Together, these findings indicate that HIV-associated oral dysbiosis is not solely driven by contemporaneous immunosuppression, but likely reflects cumulative and context-dependent factors, including chronic immune activation, mucosal immune dysfunction, and host demographic influences [19,20].

### Potential pro-inflammatory shift and loss of commensal protection in HIV-associated dysbiosis

Our study reveals an enrichment of pro-inflammatory genera, including *Fusobacterium, Alloprevotella, and Prevotella_7*, in both HIV-ART-naïve and HIV-on-ART groups. These taxa are hallmark members of the ‘red’ and ‘orange’ complexes, strongly implicated in periodontal disease and inflammation [21–23]. *Fusobacterium nucleatum*, in particular, is an important pathogen that co-aggregates with other bacteria, facilitating biofilm maturation and promoting an inflammatory environment [24]. Their persistence may reflect the chronic local and systemic inflammation commonly observed in people living with HIV and linked to non-AIDS comorbidities [9,25]. The enrichment of *Staphylococcus* in HIV-ART-naïve individuals may be concerning, as this genus includes potentially virulent pathogens such as *Staphylococcus aureus* [26], but its suppression in the ART group suggests a direct or indirect antimicrobial effect of therapy against this specific bacterium.

At the same time, the genera associated with oral health, including (*Eubacterium*) *saphenum group*, *Filifactor*, and *Aggregatibacter*, remained depleted even in ART-treated participants, suggesting incomplete immune recovery rather than an ART-driven effect. Members of the *Eubacterium* genus are often linked to health and can produce short-chain fatty acids like butyrate, which has anti-inflammatory properties [27]. The loss of these commensal organisms might signify a breakdown in ‘colonisation resistance’, i.e. the ability of a healthy microbiome to prevent pathogen overgrowth [28]. This depletion was not corrected by ART, indicating a long-term disruption of the foundational microbial community structure. This loss may create an unstable oral environment that is more susceptible to expansion/invasion by opportunists, with a corresponding increased risk of oral cavity inflammation.

### ART induces a unique transitional microbial composition

Our data illustrate that ART does not return the oral microbiome to a healthy state (as defined by control group microbial compositions). Instead, ART appears to generate its own transitional oral microbiome profile. Evidence for this includes the genus *Staphylococcus*, which was depleted in the ART-treated group relative to controls. This suggests that ART itself, or the improved immune function it facilitates, exerts specific selective pressures on the microbiome [6,29]. While the suppression of *Staphylococcus* is likely beneficial, the overall effect is a shift to a transitional state rather than a complete return to a healthy oral microbiome. These associations emphasise the importance of considering immune reconstitution and residual inflammation when interpreting microbial patterns in ART-treated individuals.

### Effects of sex and age on oral microbiome composition

This sub-analysis highlighted significant associations between sex and age, and oral microbiome composition in individuals living with HIV, particularly among those receiving ART. These findings suggest that demographic variables may shape the oral microbial ecosystem in ways that could influence susceptibility to oral complications, including opportunistic infections and chronic inflammation.

Sex-specific differences were evident in both the overall cohort and the HIV-on-ART subgroup, with each group displaying a distinct microbial composition. In both the overall cohort and HIV-on-ART group, male participants showed an enrichment of genera such as *Lactobacillus* and *Weissella.* In healthy individuals, these genera have been shown to be potentially beneficial as probiotics against periodontitis and (in the case of probiotic *Weissella cibaria* CMU) have demonstrated some antimicrobial activity against *Fusobacterium nucleatum* [30–32]. *Lactobacillus* enrichment has been reported in ART-treated HIV patients [7,20], and two probiotic-associated *Lactobacillus* species have been shown to possess antifungal activity against clinical oral *Candida* species obtained from the oral cavity of HIV/AIDS patients [33]. In contrast, the role of *Weissella* in the oral cavity, particularly in the context of HIV, remains poorly understood. Women, however, exhibited a higher relative abundance of taxa that are often associated with oral inflammation and dysbiosis, including the *Fusobacterium, (Eubacterium) nodatum* group and *Treponema* [24,34,35]. Opportunistic oral pathogens such as *Alloprevotella* and *Prevotella* [23,36,37] are also enriched in HIV ART-treated women. From our results, the persistence of *Fusobacterium* and *Treponema* in ART-treated women suggests that immune reconstitution alone may not fully correct sex-linked microbial susceptibilities, possibly reflecting behavioural, hormonal, or immunogenetic factors.

Age-related microbial shifts further complicate the ART landscape. In both the overall cohort and HIV-on-ART subgroup, increasing age was associated with an increasing abundance of *Rothia, Lactobacillus,* and *Limosilactobacillus*. Further, *Lactobacillus* and *Limosilactobacillus*, both considered as potential probiotics genera [38], were overrepresented in older individuals. In contrast, opportunistic genera *Alloprevotella* declined with age, while the decrease of a single opportunistic genus may seem beneficial, this finding occurs within a broader pattern of age-related remodelling that could contribute to long-term ecosystem instability.

Together, these findings underscore the nuanced influence of sex and age on the oral microbiome during ART treatment of HIV, shaped not only by HIV infection and ART status, but also by broader biological and host demographic factors. For example, differences in immune function, hormonal cycling, behaviour, and oral hygiene practices. These biological and/or demographic factors coupled to microbiome profiles, could potentially serve as candidate biomarkers for individualised risk assessment and targeted interventions in the personalised management of oral health for individuals living with HIV.

### Study limitation

This single-centre study established an important baseline for research in this field using an Indonesian cohort, although future, multi-site, research will be required to confirm the broader applicability of the current findings.

Given the observational design of the study, our findings represent associations rather causal measurements of the effects of ART on the oral microbiome. Additionally, the cross-sectional design of the study did not allow causal inferences to be made regarding HIV status, ART exposure, immune recovery, and microbial composition.

The overall cohort size was relatively large (245 patients). However, the HIV-ART-naïve group represented a relatively smaller fraction of the total cohort (15/245 patients), which may have limited the power of statistical comparisons involving the HIV-ART-naïve group.

Although an oral examination was conducted by specialists, certain key clinical oral health variables, such as periodontal status, caries, smoking history, and oral sexual practices were not systematically collected. Prior studies have shown that these factors can substantially influence oral microbiome composition and may attenuate HIV- or ART-associated differences [39,40]. Therefore, the absence of these variables in our dataset limited the interpretability of the observed microbial associations.

While our cross-sectional design offered a valuable snapshot of oral microbial differences across HIV-ART-naïve, HIV-on-ART, and healthy individuals, longitudinal follow-up would help clarify how oral microbial compositions transpose over time during ART.

The use of 16S rRNA sequencing profiled community composition at the genus level but does not capture species-level resolution or possible changes in the phenotypic potential between the different microbial compositions identified. Additionally, focusing on the bacterial composition of the oral microbiome may limit some of the scope of our findings, as fungi have also been shown to have a potentially significant impact on the oral microbiome of HIV versus control individuals [41].

## Conclusion

In conclusion, our findings characterise a potentially pro-inflammatory oral dysbiosis associated with HIV infection, marked by an increase of opportunistic oral pathogens and a reduction of protective commensals. Among individuals receiving ART, the oral microbiome showed a pattern that more closely resembled the HIV-negative profile, including lower levels of several opportunistic genera. However, key health-associated taxa remained depleted, suggesting that the microbial community structure in ART-treated individuals remains ‘transitional’ and distinct from that of healthy controls. Our results further suggest that targeted biological and/or demographic factors coupled to microbiome profiles, could potentially serve as candidate biomarkers for individualised risk assessment and targeted interventions in the personalised management of oral health for individuals living with HIV.

## Supplementary Material

Supplementary Figure 1 B.png Supplementary Figure 1 B.png

Supplementary Figure 1 A.png Supplementary Figure 1 A.png

Supplementary_Data_3_.docx Supplementary_Data_3_.docx

Supplementary Figure 1 C.png Supplementary Figure 1 C.png

Supplementary Data 2.docx Supplementary Data 2.docx

Supplementary Figure 1 D.png Supplementary Figure 1 D.png

Supplemental material Supplementary_Data_1.docx

## Data Availability

Due to the nature of the research, and due to legal restrictions under the Material/Data Transfer Agreement of the Ministry of Health of the Republic of Indonesia, supporting data is not available.
