# Supplementary figures and images for: Transition of oral microbiome profile in HIV-infected Indonesian patients: the role of antiretroviral therapy
Source: J Oral Microbiol. 2026 Jan 2;18(1):2609445. doi: 10.1080/20002297.2025.2609445 (PMC12777814; doi:10.1080/20002297.2025.2609445)

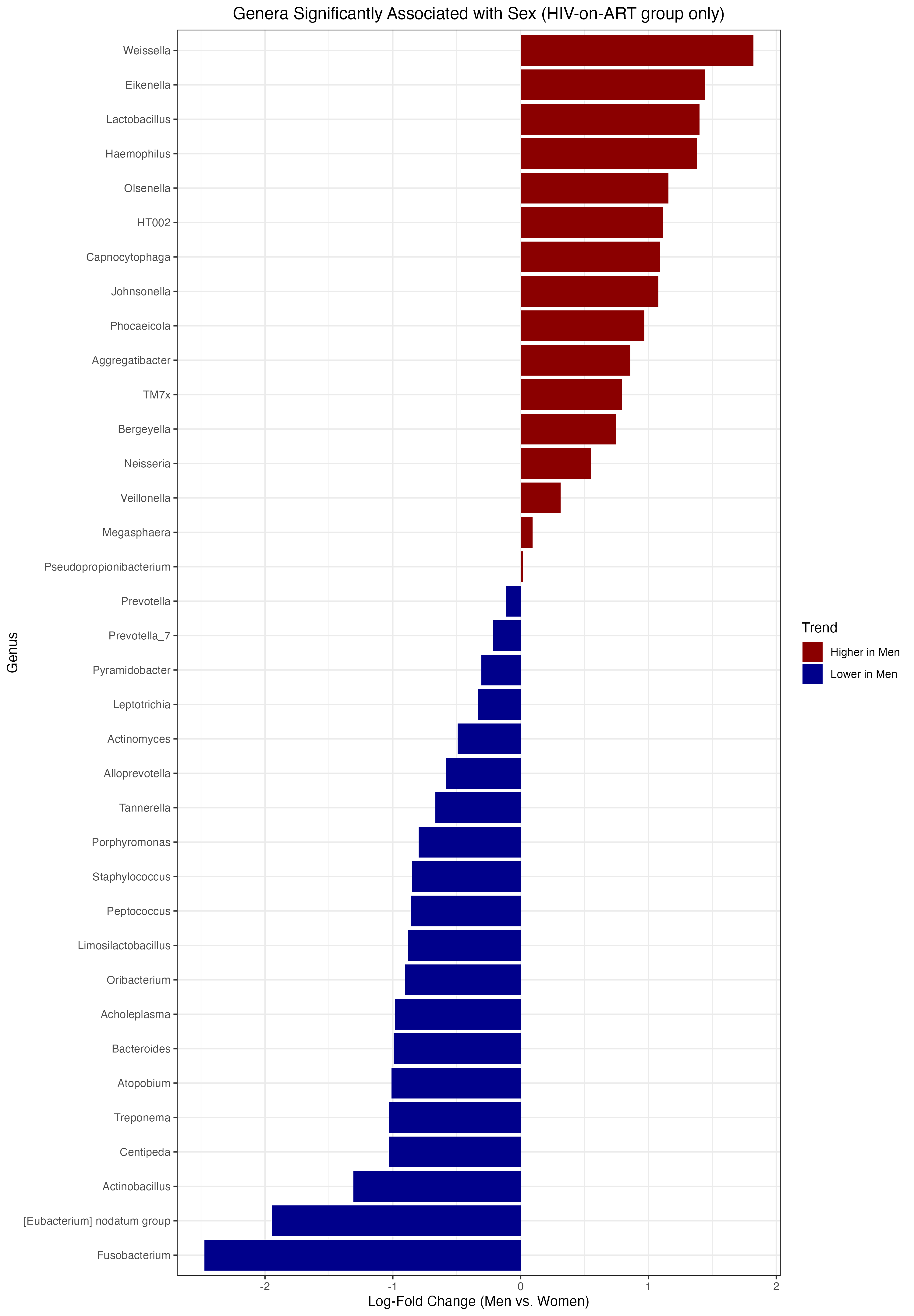

Supplement: Supplementary Figure 1 B.png [file ZJOM_A_2609445_SM8075.png]

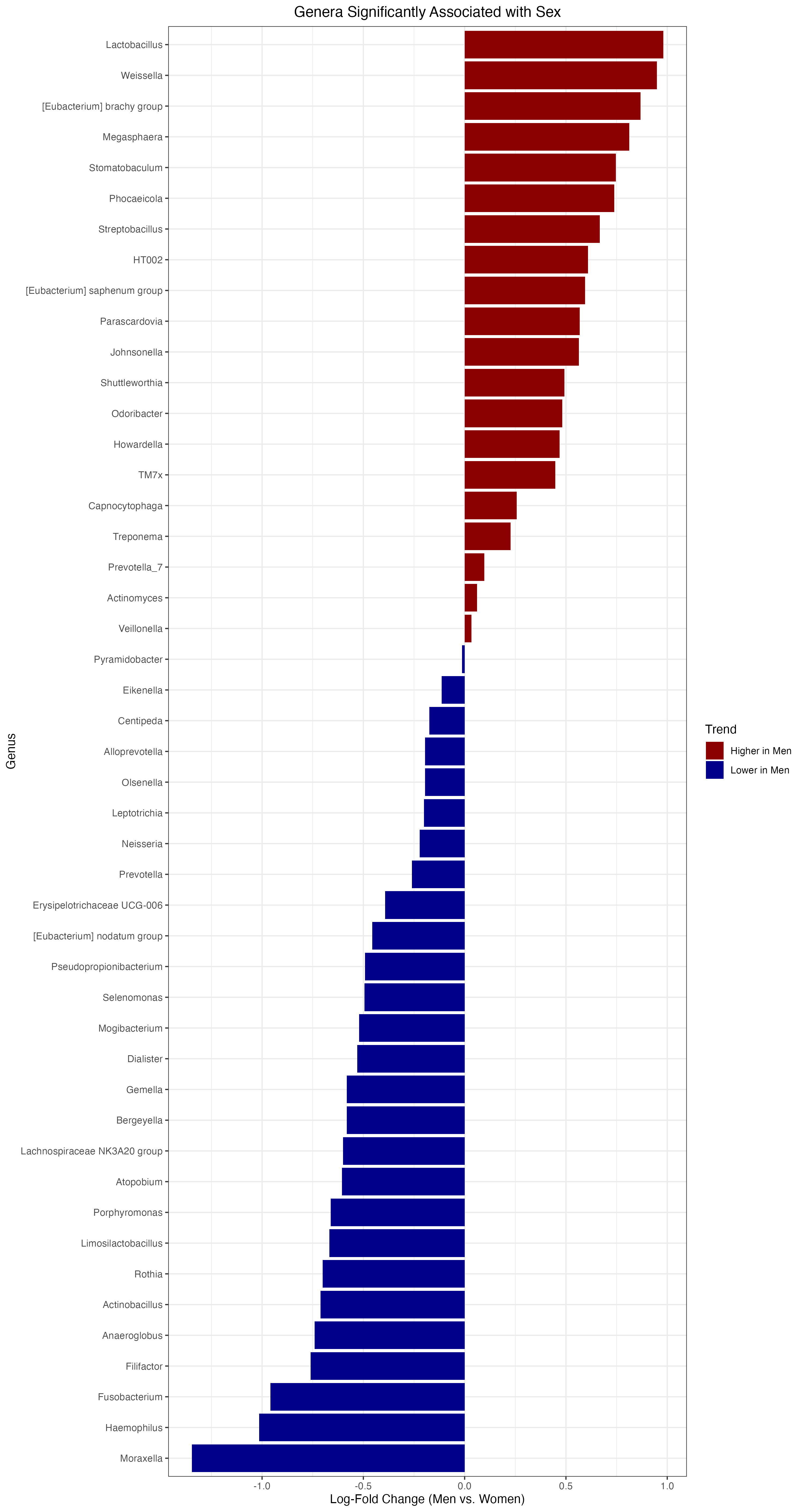

Supplement: Supplementary Figure 1 A.png [file ZJOM_A_2609445_SM8070.png]

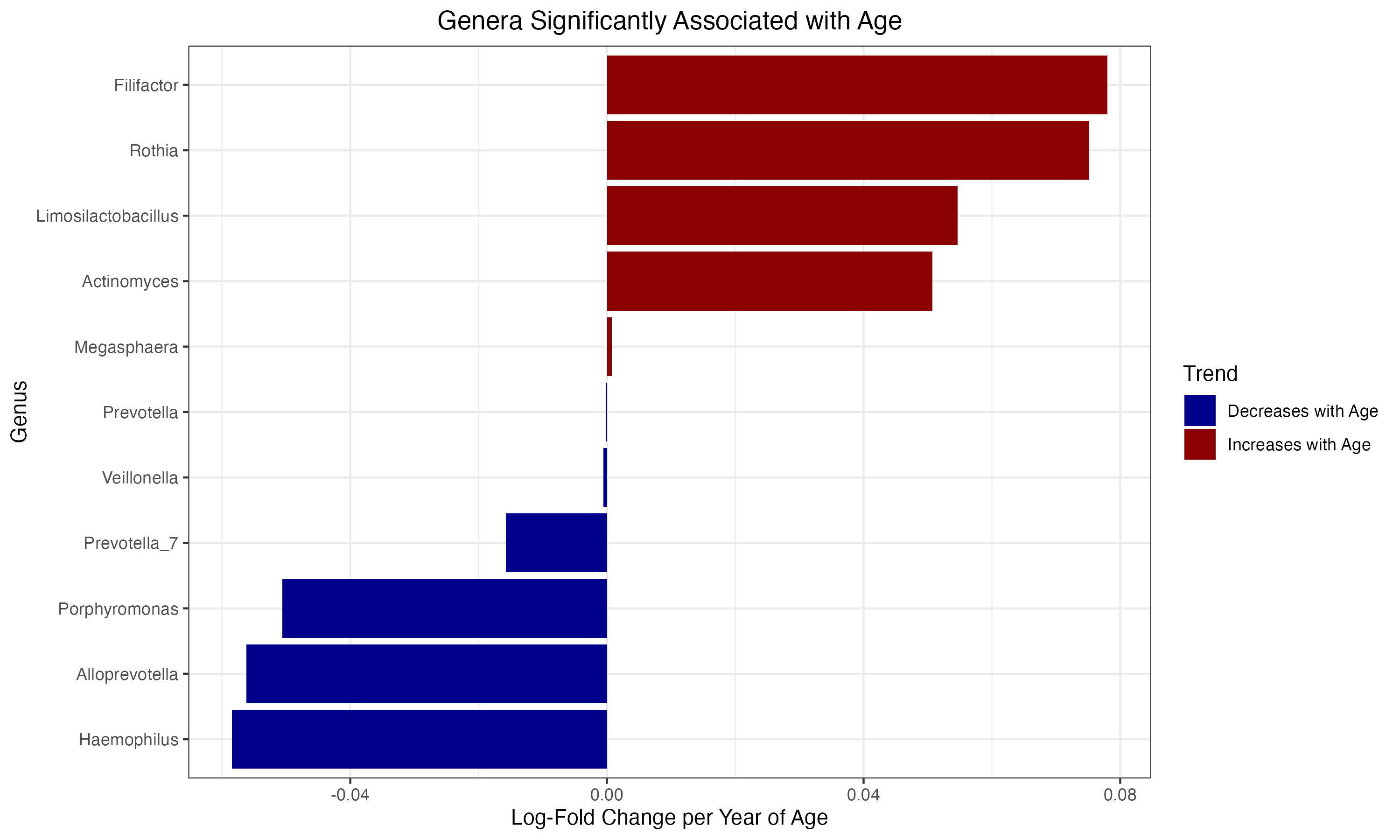

Supplement: Supplementary Figure 1 C.png [file ZJOM_A_2609445_SM8072.png]

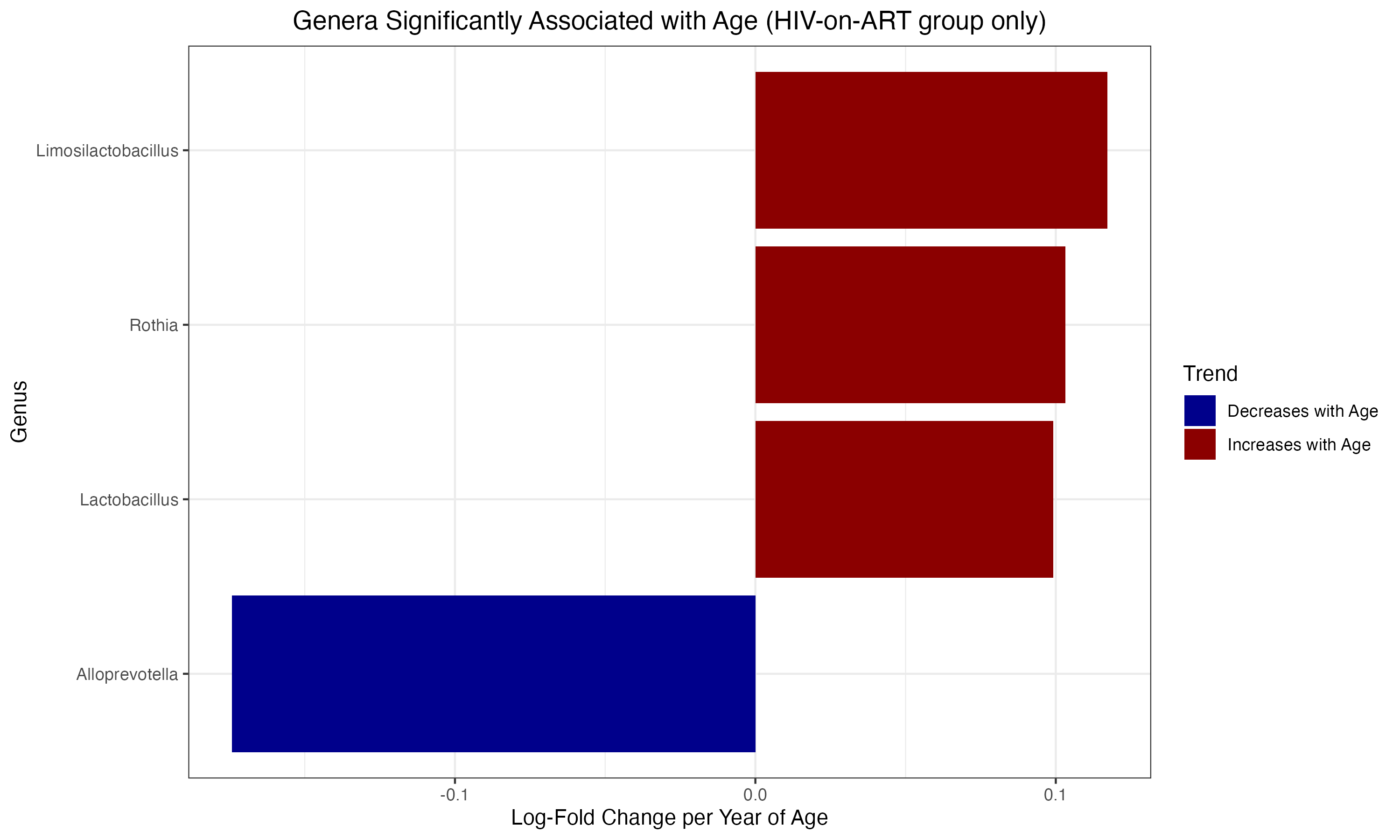

Supplement: Supplementary Figure 1 D.png [file ZJOM_A_2609445_SM8074.png]
