# Supplementary material for: Transition of oral microbiome profile in HIV-infected Indonesian patients: the role of antiretroviral therapy
Source: J Oral Microbiol. 2026 Jan 2;18(1):2609445. doi: 10.1080/20002297.2025.2609445 (PMC12777814; doi:10.1080/20002297.2025.2609445)
Supplement: Supplementary_Data_3_.docx [file ZJOM_A_2609445_SM8071.docx]

**Supplementary Data 3. PERMANOVA analysis on HIV groups only**

| **Variables** | **Univariate** | | | **Multivariate model**  **(n = 125)** | | |
| --- | --- | --- | --- | --- | --- | --- |
|  | **R^2^** | **F** | **p** | **R^2^** | **F** | **p** |
| Age | 0.021 | 2.798 | 0.001 | 0.018 | 2.319 | 0.001 |
| Sex | 0.010 | 1.249 | 0.138 | 0.010 | 1.212 | 0.168 |
| Body Mass Index | 0.009 | 1.180 | 0.207 | 0.010 | 1.204 | 0.147 |
| CD4+ T-cell count | 0.009 | 1.188 | 0.163 | 0.006 | 0.762 | 0.859 |
| Total Oral Health Habit Score | 0.006 | 0.760 | 0.866 | 0.006 | 0.742 | 0.865 |
| ART Status (naïve vs. on ART) | 0.012 | 1.548 | 0.034 | 0.010 | 1.222 | 0.178 |
