# Supplementary material for: Transition of oral microbiome profile in HIV-infected Indonesian patients: the role of antiretroviral therapy
Source: J Oral Microbiol. 2026 Jan 2;18(1):2609445. doi: 10.1080/20002297.2025.2609445 (PMC12777814; doi:10.1080/20002297.2025.2609445)
Supplement: Supplementary Data 2.docx [file ZJOM_A_2609445_SM8073.docx]

**Supplementary Data 2**

| **Oral Health Habit Score** |  |  |
| --- | --- | --- |
| Variable | Responses | Score |
| Toothbrushing frequency per day | Never | 0 |
|  | 1–2 times per day | 1 |
|  | 3 times or more per day | 2 |
| Toothbrushing before bedtime, per week | 0 | 0 |
|  | 1–3 times | 1 |
|  | 4–6 times | 2 |
|  | 7 times (every day) | 3 |
| Use of oral hygiene products (mouthwash) | No | 0 |
|  | Yes | 1 |
| Use of interdental brush or dental floss | Never | 0 |
|  | Sometimes | 1 |
|  | Always after brushing | 2 |
| History of last dental scaling (professional cleaning) | Never | 0 |
|  | More than 2 years ago | 1 |
|  | Less than 2 years ago | 2 |
|  | Less than 1 year ago | 3 |
|  | Less than 6 months ago | 4 |
| Total maximum score |  | 12 |
