# Supplementary material for: Transition of oral microbiome profile in HIV-infected Indonesian patients: the role of antiretroviral therapy
Source: J Oral Microbiol. 2026 Jan 2;18(1):2609445. doi: 10.1080/20002297.2025.2609445 (PMC12777814; doi:10.1080/20002297.2025.2609445)
Supplement: Supplemental material — Supplementary_Data_1.docx [file ZJOM_A_2609445_SM4353.docx]

**Supplementary Data 1**

**A.
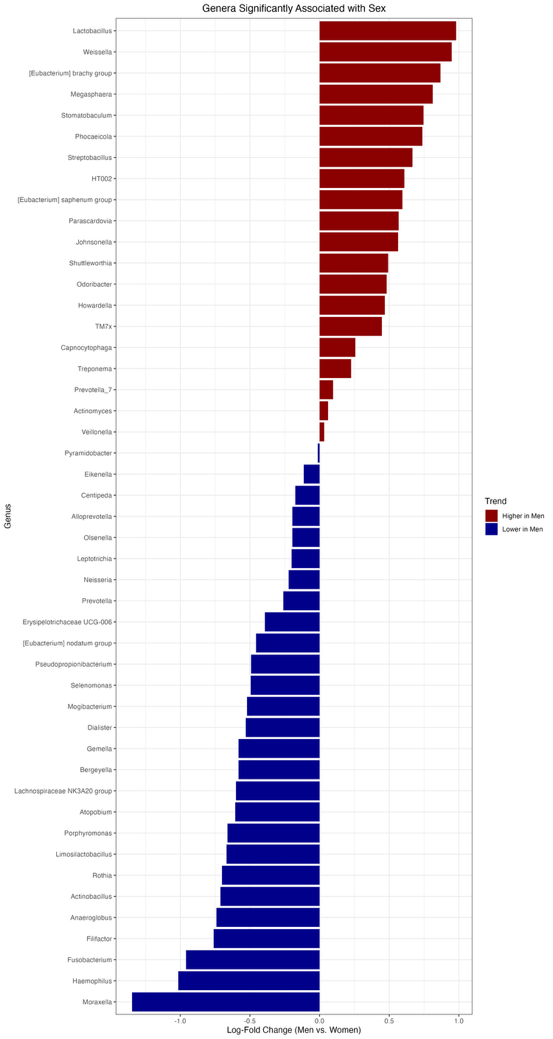
B.
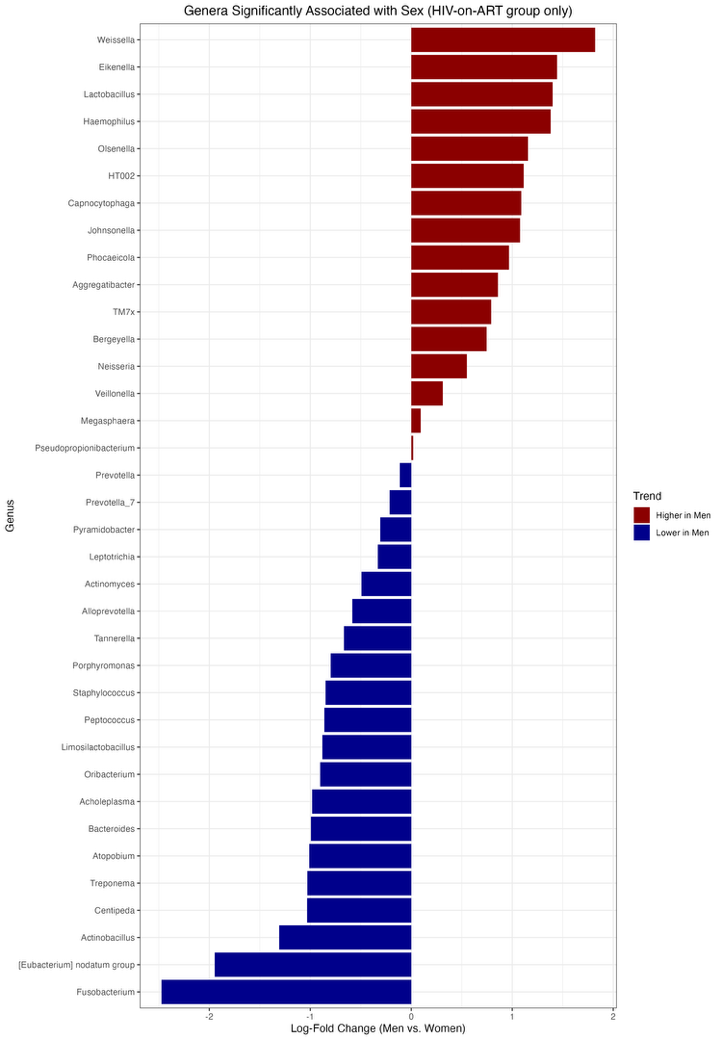
**

**C.
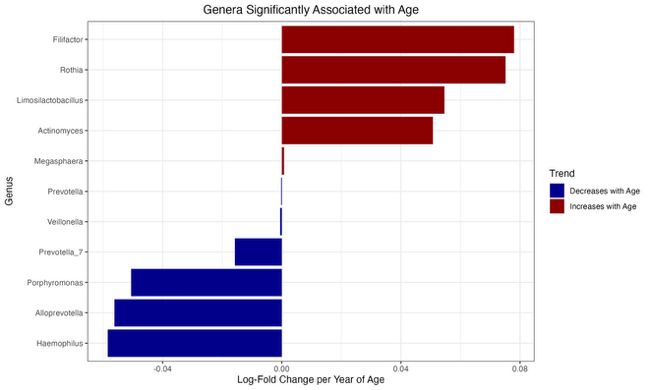
D.
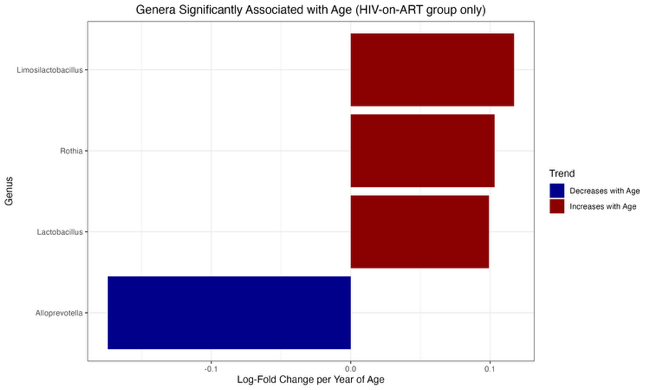
**

**Supplementary Figure 1. Differential abundance of oral bacterial genera by demographic factors.**

(A) Waterfall plot showing LFC (men vs. women) for genera with significant sex-based differences (ANCOM-BC, FDR< 0.05). (B) Waterfall plot showing LFC (men vs. women) in HIV-on-ART group for genera with significant sex-based differences (ANCOM-BC, FDR< 0.05). Red bars indicate genera enriched in men; blue bars indicate enrichment in women. (C) Waterfall plot showing LFC per year of age for genera significantly associated with aging (ANCOM-BC, FDR< 0.05). (D) Waterfall plot showing LFC per year of age in HIV-on-ART group for genera significantly associated with aging (ANCOM-BC, FDR< 0.05). Red bars indicate genera increasing with age; blue bars indicate genera decreasing with age. All genera are ranked by effect size.
